# Supplementary material for: Longitudinal analysis of body weight reveals homeostatic and adaptive traits linked to lifespan in diversity outbred mice
Source: Nat Commun. 2026 Jan 19;17:1689. doi: 10.1038/s41467-026-68392-6 (PMC12909300; doi:10.1038/s41467-026-68392-6)
Supplement: Supplementary file 1 — Supplementary Tables, Figures, and Methods [file 41467_2026_68392_MOESM1_ESM.pdf]

Supplementary Information

Longitudinal analysis of body weight reveals homeostatic and adaptive traits linked to lifespan in diversity outbred mice

G.V. Prateek[1], Zhenghao Chen[1], Kevin Wright[1], Andrea Di Francesco[1], Vladimir Jojic[1], Gary A. Churchill[2], Anil Raj[1]

[1]Calico Life Sciences LLC, South San Francisco, United States

[2]The Jackson Laboratory, Bar Harbor, United States

Supplementary Contents

Supplementary Tables **2**

ARHMM parameter estiamtes . . . 2

Body weight-derived phenotypes and defintions . . . 2

Supplementary Figures **3**

Phenotyping events, proportion of variance, and body weight trajectories . . . 3

Variational inference-based autoregressive hidden Markov model . . . 4

Influence of diet and age on the state transitions . . . 5

Influence of diet on stable maintenance of states . . . 6

Adaptation to stress is influenced by diet and age . . . 7

State occupancy association with lifespan analysis expanded . . . 8

Body weight-derived traits and its association with lifespan . . . 9

Phenotypic-genotypic correlations . . . 10

Evidence supporting mapped genetic loci . . . 11

Variational inference-based autoregressive hidden Markov model **12**

Model parameters . . . 12

Evidence-based lower bound . . . 13

Variational Bayes E-Step . . . 15

Variational Bayes M-Step . . . 16

M-Step . . . 17

Deviance information criteria . . . 18

Supplementary Tables

ARHMM parameter estiamtes

Supplemental Table 1: Estimated parameters of the variational inference-based autoregressive hidden Markov model ($y_t = \phi_0^{s_t} + \phi_1^{s_t} y_{t-1} + \epsilon^{s_t}, s_t \in \{0, 1, 2\}$).

State	Initial Probability	Transition Probability from State X			AR(0)	Error
		P(DS|X)	P(SS|X)	P(GS|X)		
Decline (X = DS)	$\pi_0 = 0.42$	0.42	0.21	0.37	$\phi_0^0 \sim \mathcal{N}(-1.6, 9.1 \times 10^{-4})$	$\epsilon^0 \sim \mathcal{N}(0, 2.00)$
Steady (X = SS)	$\pi_1 = 0.07$	0.07	0.93	≈0.00	$\phi_0^1 \sim \mathcal{N}(+0.1, 4.4 \times 10^{-6})$	$\epsilon^1 \sim \mathcal{N}(0, 0.64)$
Growth (X = GS)	$\pi_2 = 0.14$	0.14	0.12	0.74	$\phi_0^2 \sim \mathcal{N}(+1.4, 3.4 \times 10^{-5})$	$\epsilon^2 \sim \mathcal{N}(0, 1.83)$

Body weight-derived phenotypes and defintions

Supplemental Table 2: Body weight-derived traits and definitions

Phenotype	Definition
State occupancy	It is a measure of the duration of a physiological state in a given interval of time and can be used to assess the stability of a physiological system.
State transitions	It is a measure of changes in the state of a system over time. They can be described as the probability of switching from one state to itself or another state in a given interval of time.
Maximum absolute rate	It is the maximum rate of change experienced by a physiological state in a given interval of time. These frequently capture the maximum magnitude of the perturbation experienced by a system.
Longest bout	It is the longest continuous duration of a physiological state within in a given interval of time and can be used to assess the robustness to perturbations of a physiological system.
Start time	It is the time instance at which an event, such as maximum absolute rate or longest bout, was recorded. It can be useful in understanding abrupt changes in a system.

Supplementary Figures

Phenotyping events, proportion of variance, and body weight trajectories

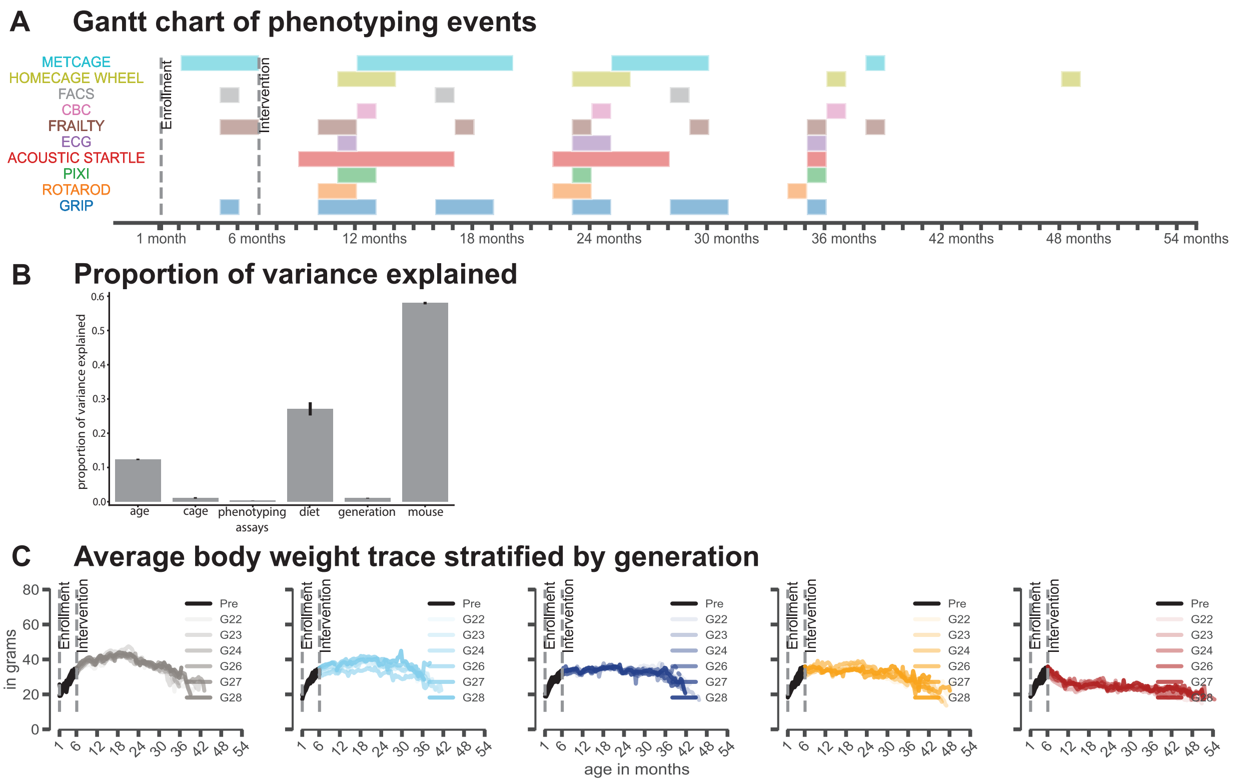

Supplemental Figure 1: **Phenotyping events, proportion of variance, and body weight**. (A) The Gantt chart illustrates the timeline for each phenotyping event in this study. Each horizontal bar represents a phenotyping event and the width of the gray bar captures the mean ± standard deviation of the duration (in months) of the phenotyping event when 95% of mice were assayed. (B) Proportion of variance explained by various factors in the study. (C) Average body weight trajectories of mice in each generation. Each panel corresponds to mice in different diets.

Variational inference-based autoregressive hidden Markov model

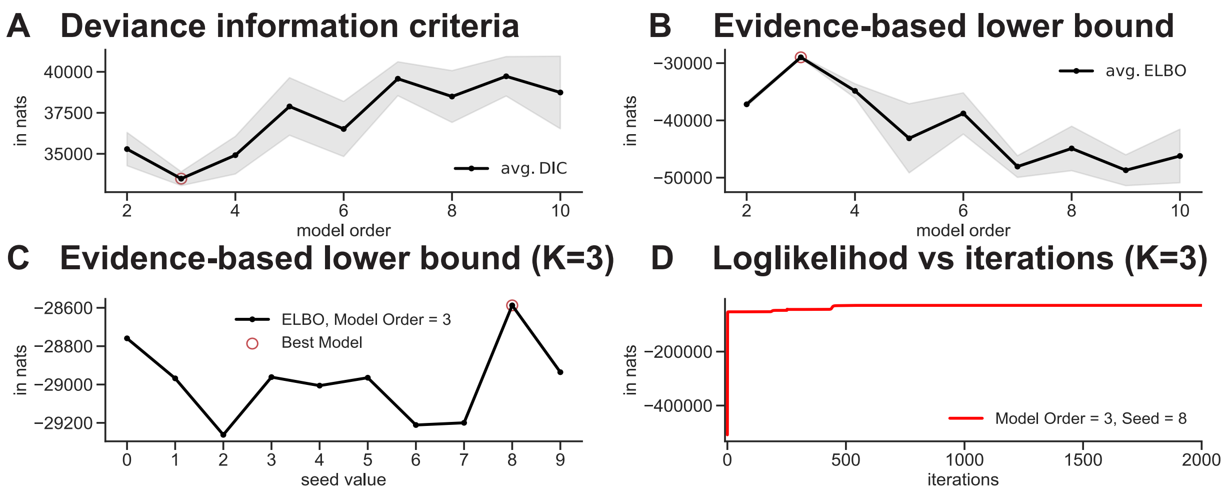

Supplemental Figure 2: **Variational inference-based autoregressive hidden Markov model**. (A) The mean value of the deviance information criteria (DIC) and standard error computed on the validation set for different model orders $K \in \{2, \ldots, 10\}$ at ten different random state initializations. Model order $K = 3$ resulted in the smallest DIC. (B) The mean value of the evidence-based lower bound (ELBO) and standard error computed on the training set for different model orders at ten different random state initializations. Once again, model order $K = 3$ resulted in the highest ELBO. (C) The ELBO of the training data for model order $K = 3$ at different random state seed values. The highest ELBO was obtained at seed value of 8 (best model). (D) Convergence of the ELBO over training iterations for the best-performing model (model order $K = 3$, seed 8) trained using the variational inference-based ARHMM approach.

Influence of diet and age on the state transitions

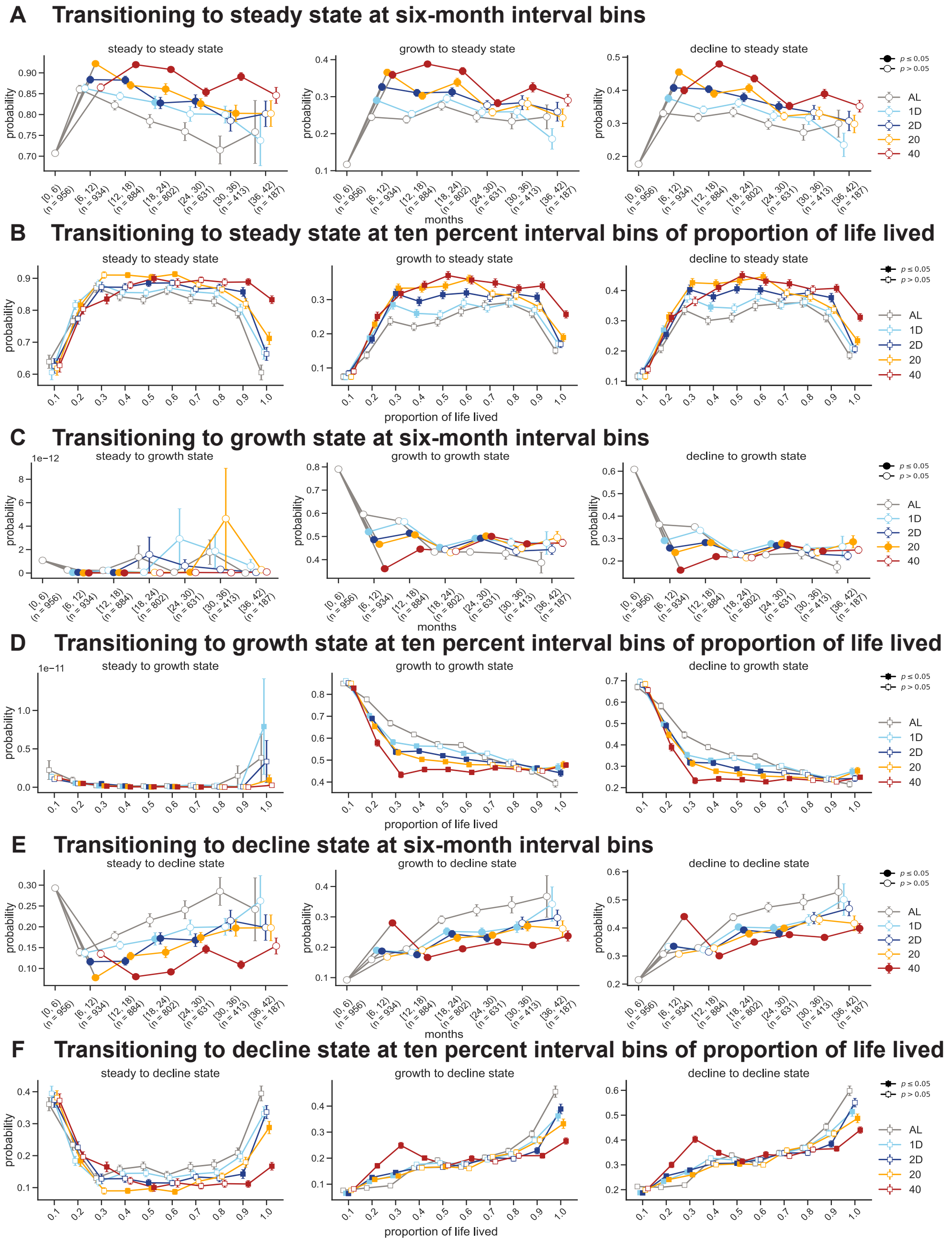

Supplemental Figure 3: **Influence of diet and age on the state transitions**. (A) and (B) Transitioning into steady state at six month and ten percent of proportion of life lived interval bins, respectively. (C) and (D) Transitioning into growth state at six month and ten percent of proportion of life lived interval bins, respectively. (E) and (F) Transitioning into decline state at six month and ten percent of proportion of life lived interval bins, respectively. In (A)-(F), solid squares or circles indicate p-values < 0.05, where the p-values were obtained by performing a two-sided Mann-Whitney test between a diet group and the AL group conditioned at the same interval bin.

Influence of diet on stable maintenance of states

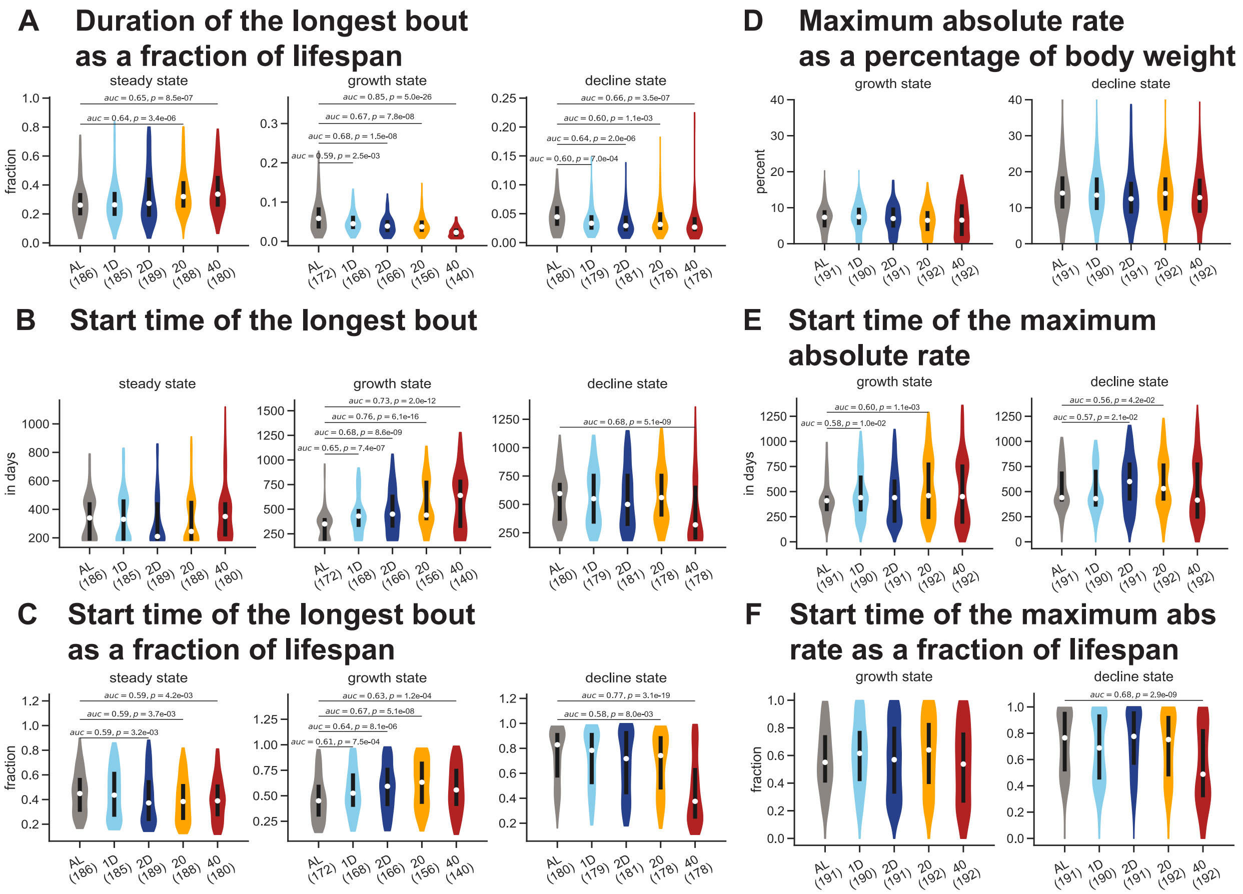

Supplemental Figure 4: **Influence of diet on stable maintenance of states**. (A) Fraction of life spent in the longest continuous bouts of growth, steady, and decline states. (B) The start time of the longest bout in growth, steady, and decline states. (C) Normalized time at which the start time of the longest bout were recorded. (D) Percentage of body weight gained and lost at the time of the maximum absolute rates of growth and decline states, respectively. (E) The time (in days) at which the maximum absolute rates of growth and decline states were recorded. (F) Normalized time at which the maximum absolute rates of growth and decline states were recorded. In (A)-(F), p-values were obtained by performing a two-sided Mann-Whitney test between two diets, with the AL diet as the reference group. The solid black line and white dot represent the interquartile range and the median, respectively.

Adaptation to stress is influenced by diet and age

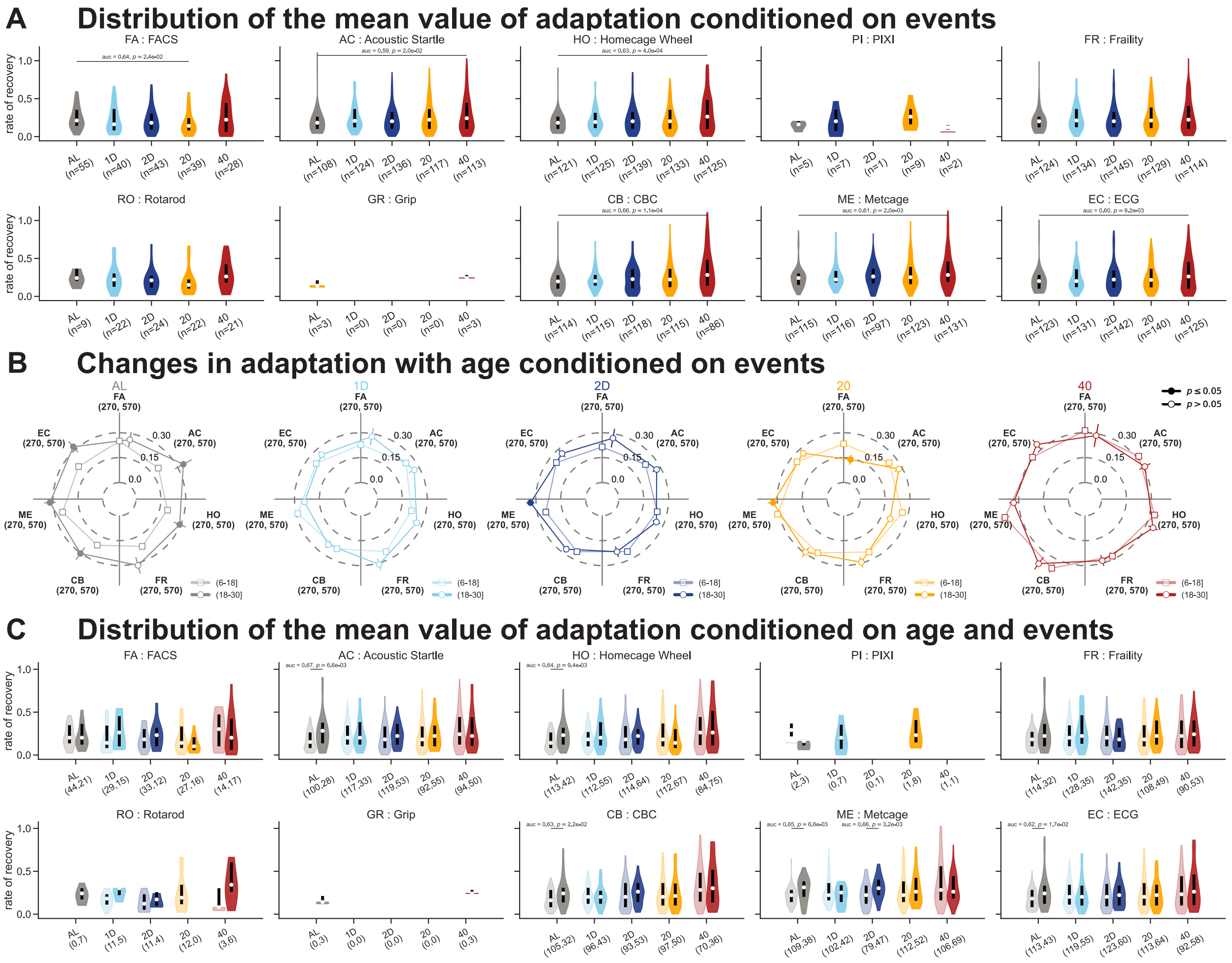

Supplemental Figure 5: **Adaptation to stress is influenced by diet and age**. (A) Violin plots of the average values of the rate of recovery, conditioned on the phenotypic assay. (B) Radar charts of the average value and the standard-errors of the rate of recovery conditioned on phenotypic assay for each diet at two non-overlapping age-bins. (C) Violin plots of the average values of the rate of recovery, conditioned on the phenotypic assay and time interval. Intervals [6–18) and [18–30) months are represented in lighter and darker shades, respectively. In (A) and (C), the number of mice for which a perturbation event was registered following a phenotypic assay in the post-intervention phase is mentioned in the parenthesis below the diet. Only a small number of perturbation events were registered after PIXI, rotarod, and grip strength phenotypic assays. The solid black line and white dot represent the interquartile range and the median, respectively. In (A)-(C), p-values were obtained by performing a two-sided Mann-Whitney test between the two time interval bins with [6–18) months as the reference group.

State occupancy association with lifespan analysis expanded

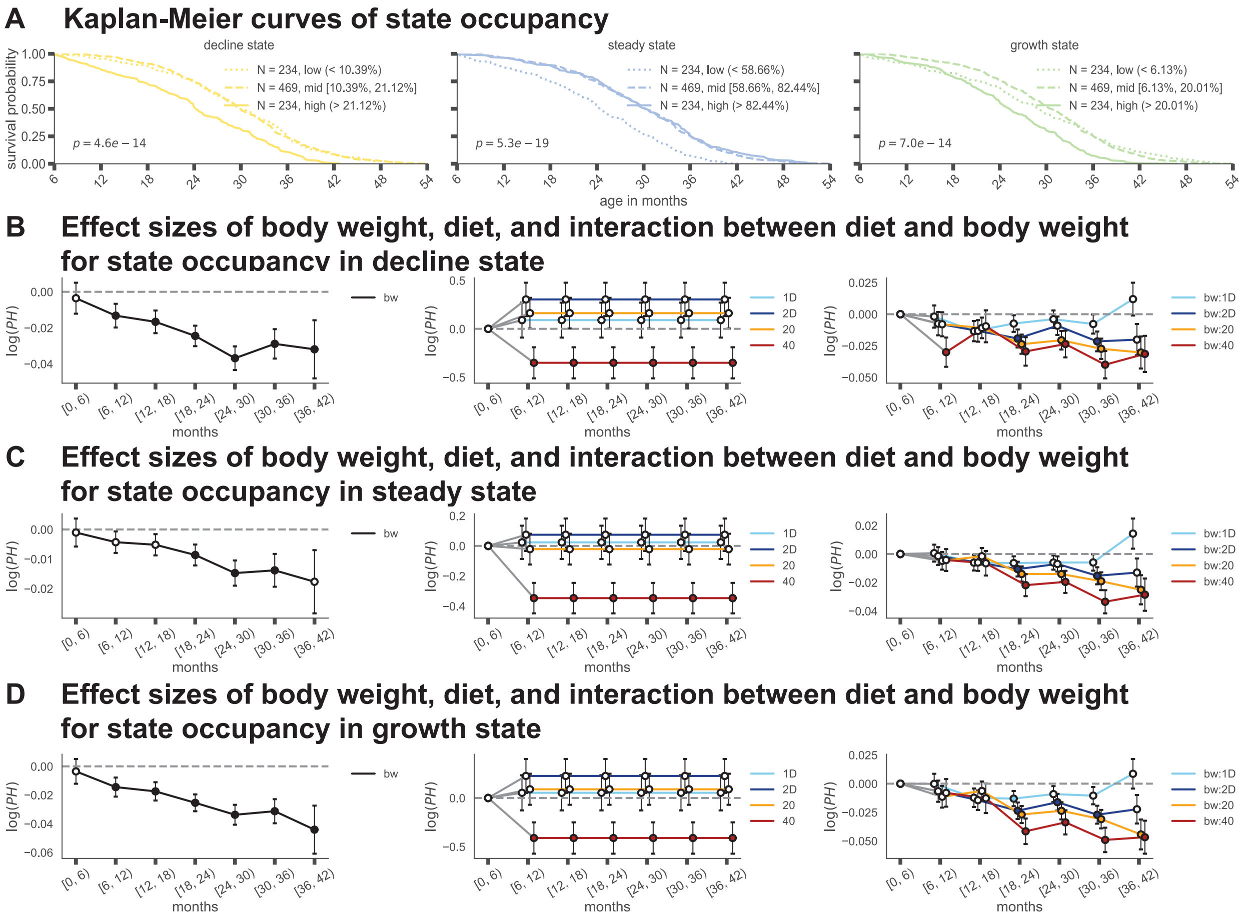

Supplemental Figure 6: **State occupancy association with lifespan analysis expanded**. (A) Kaplan-Meier curves of state occupancy phenotypes. Within each state occupancy phenotype, mice were stratified into groups as low, mid, and high, based on the distribution of the phenotypes. Mice with phenotype values greater than the upper quartile (0.75) and less than the lower quartile (0.25) were categorized as high and low, respectively, whereas the remaining were categorized as mid. Statistical significance was determined based on within-phenotype multi-log rank test for comparison within groups. (B), (C), and (D) Estimates of the effect sizes of body weight, diet, and interaction between body weight and diet, for state occupancy in decline, steady, and growth states. Solid circles indicate p-values < 0.05, where the p-values were obtained from the estimated time-varying Cox model.

Body weight-derived traits and its association with lifespan

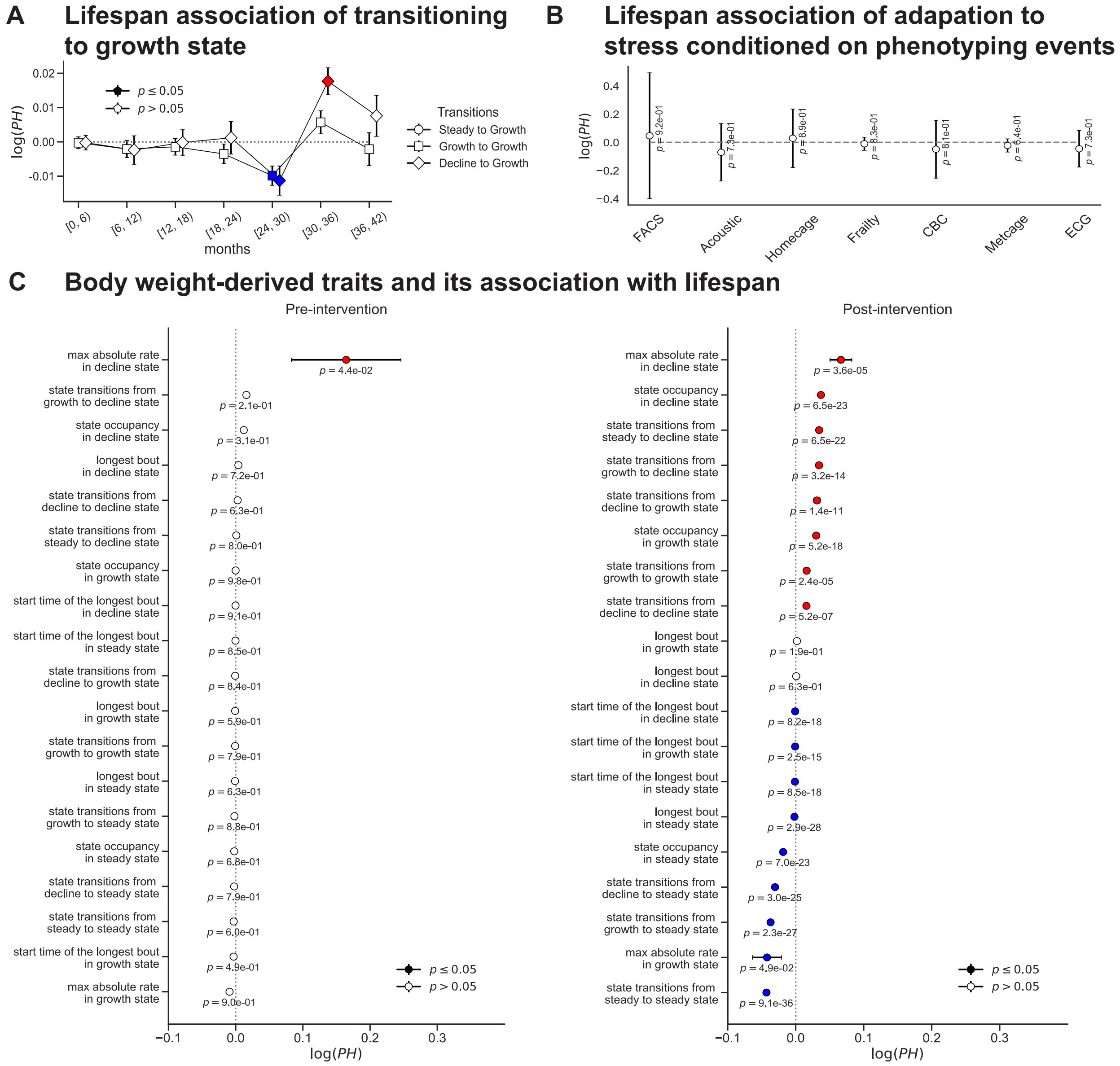

Supplemental Figure 7: **Body weight-derived traits and its association with lifespan**. (A) Effect size and standard error of transitioning to growth state and its association with lifespan at every six-month interval bins. (B) Effect size and standard error of the mean value of adaptation to stress conditioned on the phenotyping event and its association with lifespan. (C) Effect sizes and standard errors of the pre- and post-intervention body weight-derived traits that are associated with either increasing or decreasing lifespan. The traits are arranged in descending order of the effect sizes. In (A)-(C), significant associations (p-value < 0.05) were indicated with solid colors. If the effect size of the trait lies above or to the right of the gray dashed line (red filled markers), then an increase in the value of the trait decreases lifespan. Conversely, if it lies below or to the left the gray dashed line (blue filled markers), then an increase in the value of the trait increases lifespan.

Phenotypic-genotypic correlations

A Genotype-phenotype correlation matrix

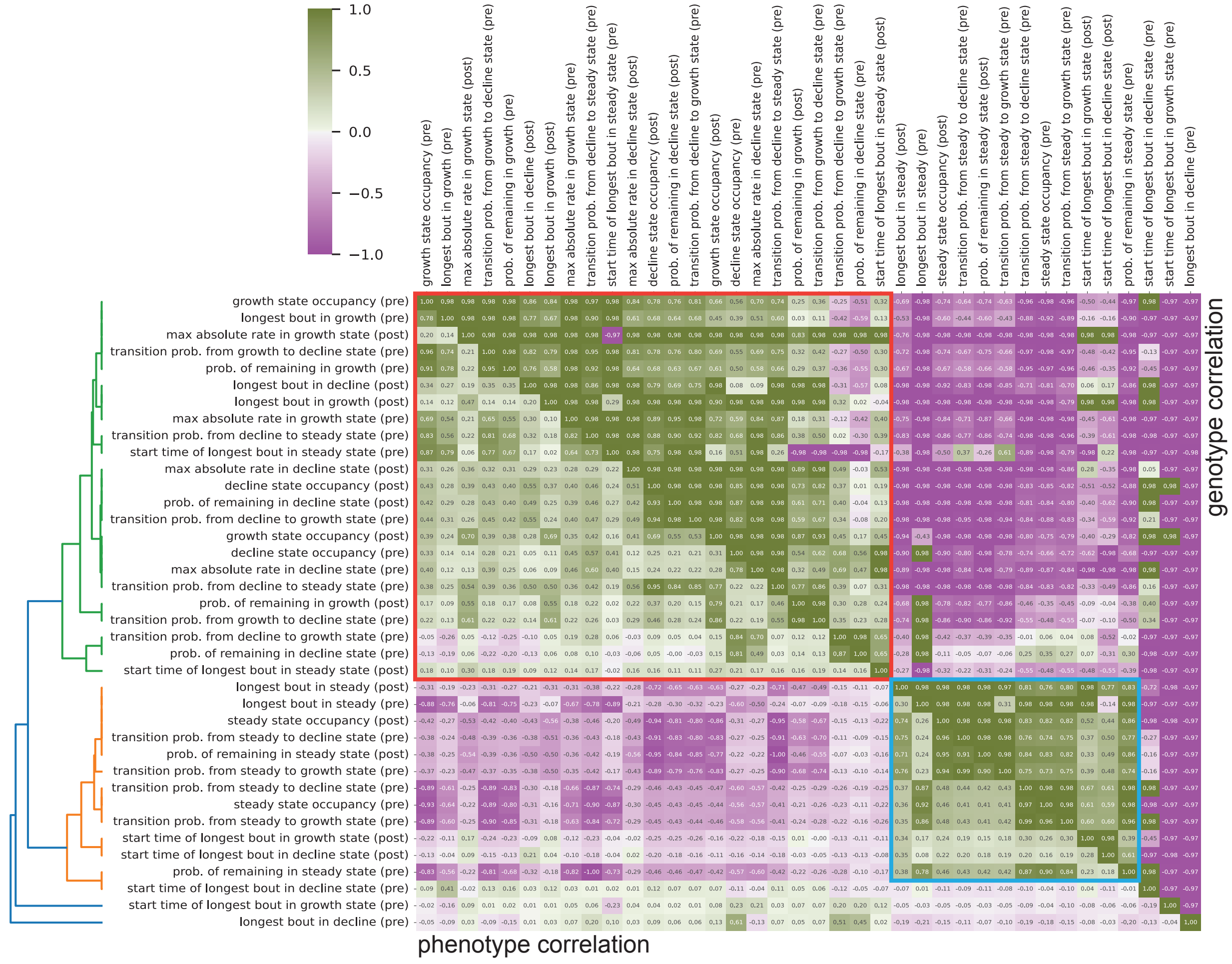

Supplemental Figure 8: **Phenotypic-genotypic correlations**. (A) Pairwise genetic (upper triangle) and phenotypic (lower triangle) correlations of the body weight-derived traits in the post-intervention phase.

Evidence supporting mapped genetic loci

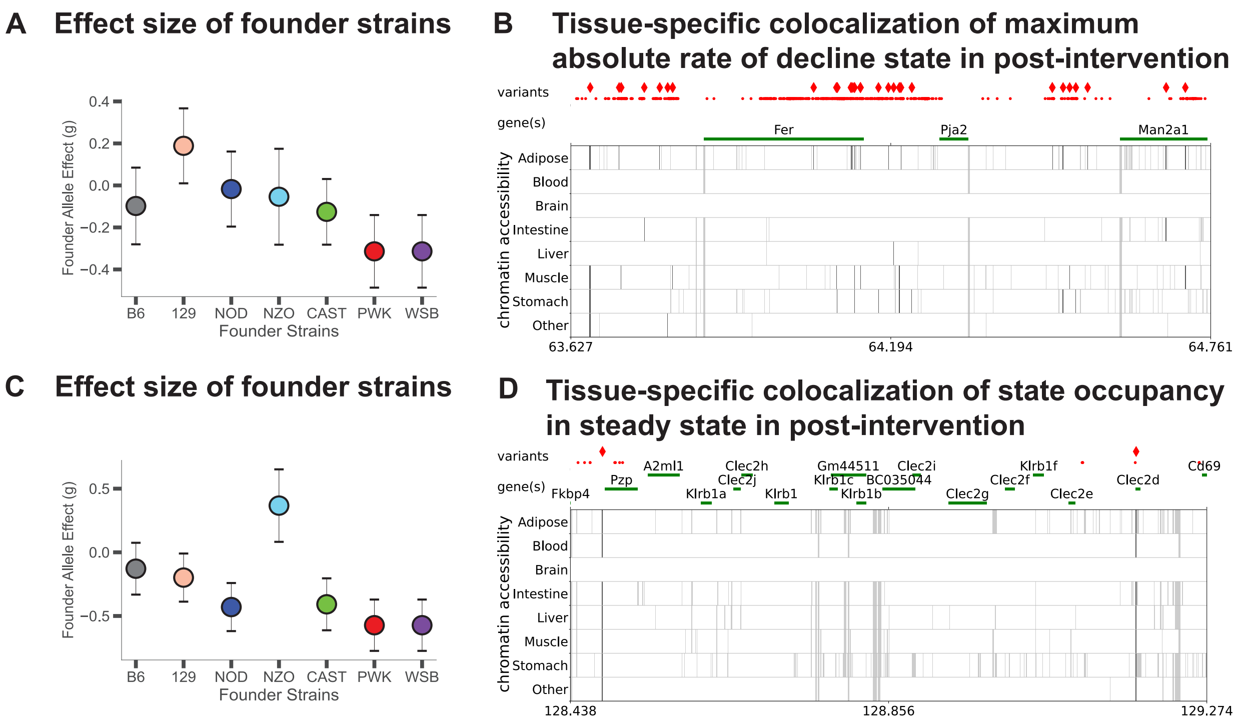

Supplemental Figure 9: **Evidence supporting mapped genetic loci**. (A) and (C) Effect size of founder allele strains observed at the variant with the highest LOD score for max absolute rate of decline and steady state occupancy, respectively, in the post-intervention phase. (B) and (D) Significant variants, colored by their founder allele pattern (FAP) group and the tissue-specific activity of regulatory elements near these variants (shown in gray). Significant variants that lie within regulatory elements are highlighted as diamonds, and regulatory elements that contain a significant variant are highlighted in black.

Variational Inference-based Autoregressive Hidden Markov Model

Model parameters

960 diversity outbred female mice were subject to 5 dietary interventions. The body weight of each mouse was measured each week (approximately) starting from one month of age. Using these high resolution temporal measurements, we wish to learn interesting physiological and developmental stages in the life of a mouse. To identify the different physiological and development stages in the life of a mouse, we develop a variational inference-based auto-regressive model.

- If M denotes the number of mice enrolled in the study, we represent the body weight trace of the m-th mouse as $\mathbf{y}_{1:T_m} = \{y_1, \ldots, y_{T_m}\}$, where T_m is the total number of body weight measurements for the m-th mouse and the body weight measurements are sorted based on the age of the mouse at the time of the measurement.

- Let $\mathbf{s}_{1:T_m} = \{s_1, \ldots, s_{T_m}\}$ denote a sequence of hidden physiological states corresponding to the body weight measurements $\mathbf{y}_{1:T_m}$, generated by a first-order Markov process, where $s_i \in \{1, \ldots, K\}$ and K is the total number of physiological states.

- Let $\boldsymbol{\pi}$ denote the initial probability vector of the first-order Markov process where,

$$\pi_j = p(s_L = j), \quad \forall 1 \leq j \leq K.$$

- Let $\mathbf{A}$ denote the state transition matrix where transition between states is governed by Markov chain whose realizations take on values $\{1, \ldots, K\}$ and the elements of the state transition matrix are given as:

$$a_{m,n} = p(s_t = n \mid s_{t-1} = m) \text{ such that } \sum_{n=1}^{K} a_{m,n} = 1, \quad \forall 1 \leq m \leq K,$$

  where $a_{m,n}$ is the probability of transitioning from $s_{t-1} = m$ to $s_t = n$.

- The dynamics of the body weight measurements at a given physiological state s_t can be defined using an auto-regressive model as:

$$y_t^{s_t} = \phi_0^{s_t} + y_{t-1}^{s_t}\phi_1^{s_t} + \ldots + y_{t-L}^{s_t}\phi_L^{s_t} + \epsilon^{s_t}, \quad \forall L < t \leq T_m,$$

where L is the lag order, $\phi_l^{s_t}$ is the auto-regressive coefficient for the l-th lag order, and $\epsilon^{s_t} \sim \mathcal{N}(0, \sigma_{s_t}^2)$ is zero mean additive white Gaussian noise.

- We assume that the mouse-specific parameters are unobserved and propose a hierarchical model on the dynamics parameters. If $\boldsymbol{\phi}_l = [\phi_{l,1}, \ldots, \phi_{l,K}]$ denotes a vector of autoregressive coefficients for the l-th lag order, then $\boldsymbol{\phi}_l \sim \mathcal{N}(\boldsymbol{\eta}_l, \boldsymbol{\Sigma}_l)$, where $\boldsymbol{\eta}_l = [\eta_{l,1}, \ldots, \eta_{l,K}]$ and $\boldsymbol{\Sigma}_l$ is a diagonal covariance matrix with diagonal elements $\{\sigma_{l,1}^2, \ldots, \sigma_{l,K}^2\}$. For notation convenience, we concatenate all auto-regressive coefficients into a long vector $\boldsymbol{\phi} = [\boldsymbol{\phi}_0^{\cdot} \ldots, \boldsymbol{\phi}_L^{]}$.

Evidence-based lower bound

The joint likelihood can be written as:

$$p(\mathbf{y}_{1:T_m}, \mathrm{s}_{1:T_m}, \boldsymbol{\phi}) = p(s_L) \prod_{t=L+1}^{T_m} p(s_t \mid s_{t-1}) P(y_t \mid y_{t-1:t-L}, s_t, \boldsymbol{\phi}) \prod_{l=0}^{L} p(\boldsymbol{\phi}_l \mid \boldsymbol{\eta}_l, \boldsymbol{\Sigma}_l). \tag{1}$$

The latent variables $\mathrm{s}_{1:T_m}$ and $\boldsymbol{\phi}$ are not observed. Therefore, we integrate the latent variables and rewrite the complete log-likelihood as:

$$\begin{aligned}
\log p(\mathbf{y}_{1:T_m}) &= \log \int_{\boldsymbol{\phi}} \sum_{\mathrm{s}_{1:T_m}} p(\mathbf{y}_{1:T_m}, \mathrm{s}_{1:T_m}, \boldsymbol{\phi}) d\boldsymbol{\phi}, \\
&= \log \int_{\boldsymbol{\phi}} \sum_{\mathrm{s}_{1:T_m}} \frac{p(\mathbf{y}_{1:T_m}, \mathrm{s}_{1:T_m}, \boldsymbol{\phi})}{q(\mathrm{s}_{1:T_m}, \boldsymbol{\phi})} q(\mathrm{s}_{1:T_m}, \boldsymbol{\phi}) d\boldsymbol{\phi}, \\
&= \log \mathbb{E}_q \left[\frac{p(\mathbf{y}_{1:T_m}, \mathrm{s}_{1:T_m}, \boldsymbol{\phi})}{q(\mathrm{s}_{1:T_m}, \boldsymbol{\phi})} \right], \\
&\geq \mathbb{E}_q \left[\log \frac{p(\mathbf{y}_{1:T_m}, \mathrm{s}_{1:T_m}, \boldsymbol{\phi})}{q(\mathrm{s}_{1:T_m}, \boldsymbol{\phi})} \right], \quad \text{(Jensen's inequality)} \\
&= \mathbb{E}_q [\log p(\mathbf{y}_{1:T_m}, \mathrm{s}_{1:T_m}, \boldsymbol{\phi})] - \mathbb{E}_q [\log q(\mathrm{s}_{1:T_m}, \boldsymbol{\phi})]. \quad \text{(Evidence-based lower bound)}
\end{aligned} \tag{2}$$

The log-likelihood is maximized when $q(\mathrm{s}_{1:T_m}, \boldsymbol{\phi}) = p(\mathrm{s}_{1:T_m}, \boldsymbol{\phi})$. However, the joint probability of $p(\mathrm{s}_{1:T_m}, \boldsymbol{\phi})$ is intractable. The goal of variational inference algorithm is to find the distribution $p(\mathrm{s}_{1:T_m}, \boldsymbol{\phi})$ from the chosen variational family that maximizes the lower bound to the log marginal likelihood. We want to optimize the evidence-based lower bound (ELBO) over a chosen space of variational distributions, to find the variational distribution closest to the true posterior distribution $p(\mathrm{s}_{1:T_m}, \boldsymbol{\phi})$. Therefore, we replace $p(\mathrm{s}_{1:T_m}, \boldsymbol{\phi})$ with $q(\mathrm{s}_{1:T_m}, \boldsymbol{\phi})$ such that $q(\mathrm{s}_{1:T_m}, \boldsymbol{\phi})$ is tractable and $p(\mathrm{s}_{1:T_m}, \boldsymbol{\phi}) \approx q(\mathrm{s}_{1:T_m}, \boldsymbol{\phi})$. Further, to perform this optimization, we assume that (a) the states and the autoregressive coefficients are independent, (b) autoregressive coefficients are independent, and (c) the transition between states is

governed by a first-order Markov process. Under these assumptions,

$$
\begin{aligned}
q(\mathrm{s}_{1:T_m}, \boldsymbol{\phi}) &= q(\mathrm{s}_{1:T_m}) q(\boldsymbol{\phi}), \\
&= q(\mathrm{s}_{1:T_m}) \prod_{l=0}^{L} q(\boldsymbol{\phi}_l \mid \tilde{\boldsymbol{\eta}}_l, \tilde{\boldsymbol{\Sigma}}_l),
\end{aligned}
$$

where $q(\boldsymbol{\phi}_l \mid \tilde{\boldsymbol{\eta}}_l, \tilde{\boldsymbol{\Sigma}}_l) \sim \mathcal{N}(\tilde{\boldsymbol{\eta}}_l, \tilde{\boldsymbol{\Sigma}}_l)$, $\tilde{\boldsymbol{\eta}}_l = [\tilde{\eta}_{l,1}, \ldots, \tilde{\eta}_{l,K}]$ is the mean, $\tilde{\boldsymbol{\Sigma}}_l$ is a diagonal covariance matrix with diagonal elements $\{\tilde{\sigma}_{l,1}^2, \ldots, \tilde{\sigma}_{l,K}^2\}$,

$$
q(\mathrm{s}_{1:T_m}) \propto \prod_{j=1}^{K} q(s_L = j)^{\delta(s_L = j)} \prod_{t=L+1}^{T} \prod_{m=1}^{K} \prod_{n=1}^{K} q(s_t = n \mid s_{t-1} = m)^{\delta(s_{t-1} = m, s_t = n)},
$$

and $\delta(\cdot)$ acts as a masking function which is set to one if the condition is true else zero. We expand the terms in the ELBO expression in (2) and rewrite it as:

$$
\begin{aligned}
\text{ELBO} = &\sum_{j=1}^{K} q(s_L = j) \log p(s_L) + \sum_{t=L+1}^{T_m} \sum_{m=1}^{K} \sum_{n=1}^{K} q(s_t = n, s_{t-1} = m) \log p(s_t \mid s_{t-1}) \\
&+ \sum_{t=L+1}^{T_m} \sum_{j=1}^{K} q(s_t = j) \langle \log p(y_t \mid y_{t-1:t-L}, s_t, \boldsymbol{\phi}) \rangle_{q(\boldsymbol{\phi})} + \sum_{l=0}^{L} q(\boldsymbol{\phi}_l \mid \tilde{\boldsymbol{\eta}}_l, \tilde{\boldsymbol{\Sigma}}_l) \log p(\boldsymbol{\phi}_l \mid \boldsymbol{\eta}_l, \boldsymbol{\Sigma}_l) \\
&- \sum_{j=1}^{K} q(s_L = j) \log q(s_L) - \sum_{t=L+1}^{T_m} \sum_{m=1}^{K} \sum_{n=1}^{K} q(s_t = n, s_{t-1} = m) \log q(s_t \mid s_{t-1}) \\
&- \sum_{l=0}^{L} q(\boldsymbol{\phi}_l \mid \tilde{\boldsymbol{\eta}}_l, \tilde{\boldsymbol{\Sigma}}_l) \log q(\boldsymbol{\phi}_l \mid \tilde{\boldsymbol{\eta}}_l, \tilde{\boldsymbol{\Sigma}}_l).
\end{aligned} \tag{3}
$$

The above expression can be further simplified as:

$$
\begin{aligned}
\text{ELBO} = &\langle \log p(s_L) - \log q(s_L) \rangle_{q(s_L)} + \sum_{t=L+1}^{T_m} \langle \log p(s_t \mid s_{t-1}) - \log q(s_t \mid s_{t-1}) \rangle_{q(s_t, s_{t-1})} \\
&+ \sum_{t=L+1}^{T_m} \langle \langle \log p(y_t \mid y_{t-1:t-L}, s_t, \boldsymbol{\phi}) \rangle_{q(\boldsymbol{\phi})} \rangle_{q(s_t)} + \sum_{l=0}^{L} \langle \log p(\boldsymbol{\phi}_l \mid \boldsymbol{\eta}_l, \boldsymbol{\Sigma}_l) - \log q(\boldsymbol{\phi}_l \mid \tilde{\boldsymbol{\eta}}_l, \tilde{\boldsymbol{\Sigma}}_l) \rangle_{q(\boldsymbol{\phi}_l)},
\end{aligned} \tag{4}
$$

where $\langle \cdot \rangle_{q(\cdot)}$ is the expectation with respect to $q(\cdot)$. Furthermore, based on the normality assumptions made on $q(\boldsymbol{\phi}_l \mid \tilde{\boldsymbol{\eta}}_l, \tilde{\boldsymbol{\Sigma}}_l)$, we get

$$
\langle \phi_{l,j} \rangle_{q(\phi_{l,j})} = \tilde{\eta}_{l,j}, \tag{5}
$$

$$
\langle \phi_{l,j}^2 \rangle_{q(\phi_{l,j})} = \tilde{\eta}_{l,j}^2 + \tilde{\sigma}_{l,j}^2. \tag{6}
$$

Substituting (5) and (6) in (4) gives the complete expression of ELBO which is given as:

$$
\begin{aligned}
\text{ELBO} = \langle \log p(s_L) - \log q(s_L)\rangle_{q(s_L)} + \sum_{t=L+1}^{T_m} \langle \log p(s_t \mid s_{t-1}) - \log q(s_t \mid s_{t-1})\rangle_{q(s_t, s_{t-1})} \\
- \sum_{t=L+1}^{T_m} \sum_{j=1}^{K} q(s_t = j)\Bigg[\frac{1}{2}\log \sigma_j^2 + \frac{1}{2\sigma_j^2}\Bigg(y_t^2 - 2y_t\tilde{\eta}_{0,j} - 2y_t \sum_{l=1}^{L} \tilde{\eta}_{l,j} y_{t-l} + \\
\tilde{\eta}_{0,j}^2 + \tilde{\sigma}_{0,j}^2 + \sum_{l=1}^{L} (\tilde{\eta}_{l,j}^2 + \tilde{\sigma}_{l,j}^2) y_{t-l}^2 + 2\sum_{l=1}^{L} \tilde{\eta}_{l,0}\tilde{\eta}_{l,j} y_{t-l} + 2\sum_{p=1}^{K} \sum_{\substack{q=1 \\ p \neq q}}^{K} \tilde{\eta}_{p,j}\tilde{\eta}_{q,j} y_{t-p} y_{t-q}\Bigg)\Bigg] \\
+ \sum_{l=0}^{L} \sum_{j=1}^{K} \frac{1}{2}\left[1 + \log \tilde{\sigma}_{l,j}^2 - \log \sigma_{l,j}^2 - \frac{\tilde{\sigma}_{l,j}^2 + (\tilde{\eta}_{l,j} - \eta_{l,j})^2}{\sigma_{l,j}^2}\right]
\end{aligned}
\tag{7}
$$

Maximizing the variational parameters of the ELBO by keeping the model parameters fixed gives us the variational E-step and M-step, and maximizing the model parameters of the ELBO by keeping the variational parameters fixed gives us the M-step. These steps are repeated until the convergence criteria is met.

Variational Bayes E-Step

In the variational Bayes E-step, the mouse-specific and model parameters are fixed and the ELBO is maximized over the mouse-specific variational marginal and joint probabilities. We used the forward-backward algorithm to compute the marginal and joint variational probabilities $q(s_t = j)$ and $q(s_t = n, s_{t-1} = m)$, respectively. The compact expression of ELBO in (2) can be rewritten in a functional form as:

$$
\begin{aligned}
\text{ELBO} &= \int d\boldsymbol{\phi} \sum_{\mathbf{s}_{1:T_m}} q(\mathbf{s}_{1:T_m}, \boldsymbol{\phi}) \log \frac{p(\mathbf{y}_{1:T_m}, \mathbf{s}_{1:T_m} \mid \boldsymbol{\phi}) p(\boldsymbol{\phi})}{q(\mathbf{s}_{1:T_m}, \boldsymbol{\phi})} \\
&= \int d\boldsymbol{\phi}\ q(\boldsymbol{\phi}) \left[\log \frac{p(\boldsymbol{\phi})}{q(\boldsymbol{\phi})} + \sum_{\mathbf{s}_{1:T_m}} q(\mathbf{s}_{1:T_m}) \log \frac{p(\mathbf{y}_{1:T_m}, \mathbf{s}_{1:T_m} \mid \boldsymbol{\phi})}{q(\mathbf{s}_{1:T_m})}\right] \\
&= \mathcal{F}(q(\boldsymbol{\phi}) q(\mathbf{s}_{1:T_m})).
\end{aligned}
\tag{8}
$$

Taking the functional derivative of (8) with respect to $q(\mathbf{s}_{1:T_m})$ gives:

$$
\log q(\mathbf{s}_{1:T_m}) = \langle \log p(\mathbf{y}_{1:T_m}, \mathbf{s}_{1:T_m} \mid \boldsymbol{\phi})\rangle_{q(\boldsymbol{\phi})} - \log \tilde{\mathcal{Z}}(\mathbf{y}_{1:T_m}) \tag{9}
$$

where $\tilde{\mathcal{Z}}(\mathbf{y}_{1:T_m})$ is a normalization constant. The normalization constant enables the feasibility of computing $q(s_t = j)$ and $q(s_t = n, s_{t-1} = m)$ using forward-backward algorithm. Unlike the hidden Markov model which requires the joint log-likelihood, the re-parameterized implementation of the forward-backward algorithm requires the expectation of the

partial log-likelihood. Moreover, based on the assumptions made on the variational parameters, we can easily compute the closed-form expression of the expectation of the partial log-likelihood with respect to $q(\boldsymbol{\phi})$ as follows:

$$\begin{aligned}\log q(s_{1:T_m}) &= \left\langle \log\left[p(s_L)\prod_{t=L+1}^{T_m} p(s_t \mid s_{t-1})p(y_t \mid y_{t-1:t-L}, s_t, \boldsymbol{\phi})\right]\right\rangle_{q(\boldsymbol{\phi})} - \log\tilde{\mathcal{Z}}(y_{1:T}) \\ &= \sum_{j=1}^{K} q(s_L = j)\log\pi_j + \sum_{t=L+1}^{T_m}\sum_{m=1}^{K}\sum_{n=1}^{K} q(s_{t-1} = m, s_t = n)\log a_{m,n} \\ &\quad + \sum_{t=L+1}^{T_m}\sum_{j=1}^{K} q(s_t = j)\langle\log p(y_t \mid y_{t-1:t-L}, \mathrm{s}_{t,j}, \boldsymbol{\phi})\rangle_{q(\boldsymbol{\phi})} - \log\tilde{\mathcal{Z}}(y_{1:T}),\end{aligned}$$

where

$$\begin{aligned}\langle\log p(y_t \mid y_{t-1:t-L}, \mathrm{s}_{t,j}, \boldsymbol{\phi})\rangle_{q(\boldsymbol{\phi})} &= -\left[\frac{1}{2}\log\sigma_j^2 + \frac{1}{2\sigma_j^2}\left(y_t^2 - 2y_t\tilde{\eta}_{0,j} - 2y_t\sum_{l=1}^{L}\tilde{\eta}_{l,j}y_{t-l} + \right.\right. \\ \tilde{\eta}_{0,j}^2 + \tilde{\sigma}_{0,j}^2 + \sum_{l=1}^{L}(\tilde{\eta}_{l,j}^2 + \tilde{\sigma}_{l,j}^2)y_{t-l}^2 &+ 2\sum_{l=1}^{L}\tilde{\eta}_{l,0}\tilde{\eta}_{l,j}y_{t-l} + 2\sum_{p=1}^{K}\left.\left.\sum_{\substack{q=1\\p\neq q}}^{K}\tilde{\eta}_{p,j}\tilde{\eta}_{q,j}y_{t-p}y_{t-q}\right)\right].\end{aligned} \tag{10}$$

The output of the forward-backward algorithm generates forward and backward probabilities, which are used to compute the variational marginal and joint probabilities for a specific mouse. The main advantage of the reparameterization step is that we can avoid computing the partial derivatives of the marginal and joint probabilities, which are often computationally intensive to solve.

Variational Bayes M-Step

In the variational Bayes M-step, the mouse-specific variational proabilities and model parameters are fixed and the ELBO is maximized over the variational model parameters.

- Variational initial probability $q(s_L)$: To get $q(s_L)$, we solve the following optimization problem:

$$\frac{\partial}{\partial q(s_L)}\left[\sum_{j=1}^{K} q(s_L)\log p(s_L) - \sum_{j=1}^{K} q(s_L)\log q(s_L) + \lambda\left(\sum_{j=1}^{K} q(s_L) - 1\right)\right] = 0.$$

On solving, we get:

$$q(s_L = j) = \frac{p(s_L = j)}{\sum_{j=1}^{K} p(s_L = j)} \quad \forall j \in \{1, \ldots K\}. \tag{11}$$

- Variational transition probability $q(s_t \mid s_{t-1})$: To get $q(s_t \mid s_{t-1})$, we solve the following optimization problem:

$$\frac{\partial}{\partial \tilde{a}_{m,n}}\Bigg[\sum_{t=L+1}^{T_m}\sum_{n=1}^{K}\sum_{m=1}^{K} q(s_t = n, s_{t-1} = m)\log a_{m,n} - \sum_{t=L+1}^{T_m}\sum_{n=1}^{K}\sum_{m=1}^{K} q(s_t = n, s_{t-1} = m)\log \tilde{a}_{m,n} + \lambda\Big(\sum_{n=1}^{K}\tilde{a}_{m,n} - 1\Big)\Bigg] = 0.$$

On solving, we get:

$$q(s_t = n \mid s_{t-1} = m) = \tilde{a}_{m,n} = \frac{\sum_{t=L+1}^{T_m} q(s_t = n, s_{t-1} = m)}{\sum_{t=L+1}^{T_m}\sum_{n=1}^{K} q(s_t = n, s_{t-1} = m)}, \quad \forall m, n \in \{1, \dots K\}. \tag{12}$$

- Variational variance $\tilde{\sigma}^2_{l,j}$:

$$\frac{1}{\tilde{\sigma}^2_{0,j}} = \frac{1}{\sigma^2_{0,j}} + \frac{1}{\sigma^2_j}\sum_{t=L+1}^{T_m} q(s_t = j), \quad \forall j \in \{1, \dots K\}, \tag{13}$$

$$\frac{1}{\tilde{\sigma}^2_{l,j}} = \frac{1}{\sigma^2_{l,j}} + \frac{1}{\sigma^2_j}\sum_{t=L+1}^{T_m} q(s_t = j) y^2_{t-l}, \quad \forall j \in \{1, \dots K\}, l \in \{1, \dots, L\}. \tag{14}$$

- Variational mean $\tilde{\eta}_{l,j}$:

$$\tilde{\eta}_{0,j} = \frac{\frac{\eta_{0,j}}{\sigma^2_{0,j}} + \frac{1}{\sigma^2_s}\sum_{t=L+1}^{T_m} q(s_t = j)(y_t - \sum_{l=1}^{L}\tilde{\eta}_{l,j} y_{t-l})}{\frac{1}{\sigma^2_{0,j}} + \frac{1}{\sigma^2_j}\sum_{t=L+1}^{T_m} q(s_t = j)}, \quad \forall j \in \{1, \dots K\}, \tag{15}$$

$$\tilde{\eta}_{l,j} = \frac{\frac{\eta_{l,j}}{\sigma^2_{l,j}} + \frac{1}{\sigma^2_s}\sum_{t=L+1}^{T_m} q(s_t = j) y_{t-l}(y_t - \tilde{\eta}_{0,j} - \frac{1}{2}\sum_{p=1, p\neq l}^{L}\tilde{\eta}_{p,j} y_{t-p})}{\frac{1}{\sigma^2_{0,j}} + \frac{1}{\sigma^2_j}\sum_{t=L+1}^{T_m} q(s_t = j) y^2_{t-l}}, \quad \forall j \in \{1, \dots K\}, \forall l \in \{1, \dots, L\}. \tag{16}$$

M-Step

In the M-step, the mouse-specific variational parameters are fixed and the ELBO is maximized over model parameters. We used subscript i to indicate the variational parameters from the i-th mouse.

- Initial probability $p(s_L)$:

$$p(s_L = j) = \frac{\sum_{i=1}^{M} q_i(s_L = j)}{\sum_{i=1}^{M}\sum_{j=1}^{K} q_i(s_L = j)}, \quad \forall j \in \{1, \dots K\}. \tag{17}$$

- Transition probability $p(s_t \mid s_{t-1})$:

$$p(s_t = n \mid s_{t-1} = m) = a_{m,n} = \frac{\sum_{i=1}^{M} \sum_{t=L+1}^{T_m} [\tilde{a}_{m,n}]_i q_i(s_{t-1} = m)}{\sum_{i=1}^{M} \sum_{t=L+1}^{T} \sum_{n=1}^{K} [\tilde{a}_{m,n}]_i q_i(s_{t-1} = m)} \quad \forall m, n \in \{1, \dots K\}. \tag{18}$$

- Error variance σ_j^2

$$\sigma_j^2 = \frac{\sum_{i=1}^{M} \sum_{t=L+1}^{T_m} q_i(s_t = j)[\psi_{j,t}]_i}{\sum_{i=1}^{M} \sum_{t=L+1} q_i(s_t = j)}, \quad \forall j \in \{1, \dots K\}, \tag{19}$$

where

$$[\psi_{j,t}]_i = \Bigg([y_t^2]_i - 2[y_t]_i[\tilde{\eta}_{0,j}]_i - 2[y_t]_i \sum_{l=1}^{L} [\tilde{\eta}_{l,j}]_i[y_{t-l}]_i[\tilde{\eta}_{0,j}^2]_i + [\tilde{\sigma}_{0,j}^2]_i + \sum_{l=1}^{L} ([\tilde{\eta}_{l,j}^2]_i + [\tilde{\sigma}_{l,j}^2]_i)[y_{t-l}^2]_i + 2\sum_{l=1}^{L} [\tilde{\eta}_{l,0}]_i[\tilde{\eta}_{l,j}]_i[y_{t-l}]_i + 2\sum_{p=1}^{K} \sum_{\substack{q=1 \\ p \neq q}}^{K} [\tilde{\eta}_{p,j}]_i[\tilde{\eta}_{q,j}]_i[y_{t-p}]_i[y_{t-q}]_i \Bigg).$$

- Auto-regressive coefficient parameters $(\eta_{l,j}, \sigma_{l,j}^2)$:

$$\eta_{l,j} = \frac{1}{M} \sum_{i=1}^{M} [\tilde{\eta}_{l,j}]_i, \quad \forall j \in \{1, \dots K\}, \forall l \in \{1, \dots, L\}, \tag{20}$$

$$\sigma_{l,j}^2 = \frac{1}{M} \sum_{i=1}^{M} [\tilde{\sigma}_{l,j}^2]_i + ([\tilde{\eta}_{l,j}]_i - \eta_{l,j})^2, \quad \forall j \in \{1, \dots K\}, \forall l \in \{1, \dots, L\}. \tag{21}$$

Deviance information criteria

We used deviance information criteria (DIC) to select the model order for the variational-inference based auto-regressive hidden Markov model (**?**). The DIC combines model complexity and fit, where the model complexity is obtained by taking difference between the posterior mean of the deviance and the deviance calculated at the posterior mean $\tilde{\boldsymbol{\phi}}$, and fit is obtained from the log-likelihood computed at the posterior mean $\tilde{\boldsymbol{\phi}}$. The DIC is expressed as:

$$\text{DIC} = 2p_D - 2\log p(\mathbf{y} \mid \boldsymbol{\phi}),$$

where

$$p_D = -2 \int q(\boldsymbol{\phi}) \log \frac{q(\boldsymbol{\phi})}{p(\boldsymbol{\phi})} d\boldsymbol{\phi} + 2 \log \frac{q(\tilde{\boldsymbol{\phi}})}{p(\tilde{\boldsymbol{\phi}})}.$$

Based on the assumptions made about the model and variational parameters,

$$\log p(\boldsymbol{\phi} \mid \boldsymbol{\eta}, \boldsymbol{\sigma}^2) = -\frac{1}{2}\sum_{l=0}^{L}\sum_{j=1}^{K}\left[\log(2\pi\sigma_{l,j}^2) + \frac{1}{\sigma_{l,j}^2}(\phi_{l,j} - \eta_{l,j})^2\right] \tag{22}$$

$$\log q(\boldsymbol{\phi} \mid \tilde{\boldsymbol{\eta}}, \tilde{\boldsymbol{\sigma}}^2) = -\frac{1}{2}\sum_{l=0}^{L}\sum_{j=1}^{K}\left[\log(2\pi\tilde{\sigma}_{l,j}^2) + \frac{1}{\tilde{\sigma}_{l,j}^2}(\phi_{l,j} - \tilde{\eta}_{l,j})^2\right]. \tag{23}$$

Using (22) and (23), p_D can be simplified as:

$$p_D = \sum_{l=0}^{L}\sum_{j=1}^{K}\left\{\left[\frac{1}{\sigma_{l,j}^2}(\tilde{\sigma}_{l,j}^2 + (\eta_{l,j} - \tilde{\eta}_{l,j})^2)\right] - \left[\frac{1}{\tilde{\sigma}_{l,j}^2}(\eta_{l,j} - \tilde{\eta}_{l,j})^2\right]\right\}.$$

We computed the deviance information criteria at ten different seed values for each model order. The model order with the smallest deviance information criteria is considered as the best model order. To select the best seed value at a given model order, we computed the ELBO and choose the seed value with the highest ELBO.
